# Pharmacogenomics of Dementia: Personalizing the Treatment of Cognitive and Neuropsychiatric Symptoms

**DOI:** 10.3390/genes14112048

**Published:** 2023-11-06

**Authors:** Barbara Vuic, Tina Milos, Lucija Tudor, Matea Nikolac Perkovic, Marcela Konjevod, Gordana Nedic Erjavec, Vladimir Farkas, Suzana Uzun, Ninoslav Mimica, Dubravka Svob Strac

**Affiliations:** 1 Laboratory for Molecular Neuropsychiatry, Division of Molecular Medicine, Rudjer Boskovic Institute, 10000 Zagreb, Croatia; barbara.vuic@irb.hr (B.V.); tina.milos@irb.hr (T.M.); lucija.tudor@irb.hr (L.T.); mnikolac@irb.hr (M.N.P.); marcela.konjevod@irb.hr (M.K.); gnedic@irb.hr (G.N.E.); vladimir.farkas@irb.hr (V.F.); 2 Department for Biological Psychiatry and Psychogeriatry, University Hospital Vrapce, 10000 Zagreb, Croatia; suzana.uzun@gmail.com (S.U.); ninoslav.mimica@bolnica-vrapce.hr (N.M.); 3 School of Medicine, University of Zagreb, 10000 Zagreb, Croatia

**Keywords:** dementia, genetics, pharmacogenomics, cognitive impairment, neuropsychiatric symptoms, drug response, adverse drug effects, drug interactions

## Abstract

Dementia is a syndrome of global and progressive deterioration of cognitive skills, especially memory, learning, abstract thinking, and orientation, usually affecting the elderly. The most common forms are Alzheimer’s disease, vascular dementia, and other (frontotemporal, Lewy body disease) dementias. The etiology of these multifactorial disorders involves complex interactions of various environmental and (epi)genetic factors and requires multiple forms of pharmacological intervention, including anti-dementia drugs for cognitive impairment, antidepressants, antipsychotics, anxiolytics and sedatives for behavioral and psychological symptoms of dementia, and other drugs for comorbid disorders. The pharmacotherapy of dementia patients has been characterized by a significant interindividual variability in drug response and the development of adverse drug effects. The therapeutic response to currently available drugs is partially effective in only some individuals, with side effects, drug interactions, intolerance, and non-compliance occurring in the majority of dementia patients. Therefore, understanding the genetic basis of a patient’s response to pharmacotherapy might help clinicians select the most effective treatment for dementia while minimizing the likelihood of adverse reactions and drug interactions. Recent advances in pharmacogenomics may contribute to the individualization and optimization of dementia pharmacotherapy by increasing its efficacy and safety via a prediction of clinical outcomes. Thus, it can significantly improve the quality of life in dementia patients.

## 1. Introduction

Pharmacotherapy of dementia is partially effective in only some individuals, with side effects, drug interactions, intolerance, and non-compliance occurring in the majority of dementia patients. Interindividual variability in drug response among dementia patients is largely due to genetic variations, which could influence the activity or availability of drug-metabolizing enzymes, receptors, channels, transporters, and other proteins involved in drug pharmacokinetics and pharmacodynamics [1]. Pharmacokinetics refers to the variability in the drug’s absorption, distribution, metabolism, and elimination (ADME) that modulates the delivery or removal of drugs and their metabolites at their action sites. On the other hand, pharmacodynamics refers to variability in the drug action dependent on the interaction of the active drug with its target molecules, such as receptors, ion channels, and enzymes, and can also affect therapeutic response and drug side effects. Different studies aimed to identify genetic variants that could predict patients who may optimally benefit from specific, individually tailored treatment. Both pharmacogenetics and pharmacogenomics, as rapidly growing fields with huge potential in drug discovery and personalized medicine, address interindividual variations in DNA sequence affecting drug efficacy and toxicity in order to optimize the pharmacotherapy based on the patient individual genetic signature. Whereas pharmacogenetics generally refers to the variations in a single or several genes influencing the response to drugs, pharmacogenomics addresses genome-wide alterations and the mutual interaction of many genes affecting drug efficacy and safety.

The development of pharmacogenomics as an interdisciplinary large-scale systematic approach has been reinforced by the introduction of genomic techniques, such as genotyping, gene sequencing, gene expression, genetic epidemiology, transcriptomics, proteomics, metabolomics and bioinformatics, and other multiplex assay technologies, which allow deeper assessment of disease mechanisms, potential drug targets and metabolism, or associated pathway components [2]. However, the application of pharmacogenomics in dementia patients is very challenging since dementia is a complex disorder represented not only by cognitive decline but also by behavioral and neuropsychiatric symptoms, as well as progressive functional deterioration, in which more than 200 different genes associated with the dementia pathogenesis, drug mechanism of action, phase I and phase II metabolism reactions, transporters, and concomitant pathologies might be involved [3,4]. A complex clinical picture of dementia usually requires simultaneous therapy with several different drugs, targeting both cognitive and neuropsychiatric symptoms. Specifically, patients with dementia typically receive 6–10 different drugs per day, including conventional anti-dementia drugs, antidepressants, antipsychotics, anxiolytics, anticonvulsants, and also other types of drugs (antihypertensive drugs, diuretics, statins, anti-histaminics, anti-inflammatory, and antidiabetic drugs), for treating various comorbid and somatic disorders in the elderly [5].

Therefore, optimization of therapy in dementia patients is a major goal to which pharmacogenomics could contribute by improving patient stratification, resulting in more effective therapy and reduced drug adverse effects. In this review, we summarize current knowledge on molecular mechanisms of dementia, the most relevant associated genes, as well as genes involved in the activity or availability of drugs commonly used for the management of both cognitive and neuropsychiatric symptoms of dementia. We also discuss the importance of pharmacogenomics studies in the search for predictive strategies and new and effective medications for dementia.

## 2. Dementia

Dementia is a complex condition that involves the interaction of various factors such as genetics, epigenetics, metabolic and vascular health abnormalities, as well as various environmental influences, ultimately resulting in the death of brain cells. This multifaceted syndrome represents a significant healthcare problem around the world and ranks as the foremost cause of disability among the elderly. The weight of dementia extends its impact not only on individuals but also on their caregivers and healthcare systems, given the profound cognitive and functional impairments it entails. A concise medical history, as well as neurological and cognitive examinations, are necessary in order to evaluate possible dementia, with the patient’s history gathered from both the individual and a family member or friend playing a crucial role in this process.

The cognitive assessment aims to determine the presence and characteristics of cognitive deficits and often utilizes screening tools such as the Montreal Cognitive Assessment (MoCA) or the Mini-Mental State Examination (MMSE), while the neurologic examination assesses neurocognitive problems (agnosia, aphasia, and apraxia) and unusual behaviors associated with specific types of dementia [6]. The standard assessment includes blood tests and neuroimaging to identify potential causes of dementia, with specialized neuropsychological testing being required in specific instances. Advanced diagnostic tools, such as positron emission tomography (PET) scans and cerebrospinal fluid testing, can provide valuable information in atypical or diagnostically challenging cases, while genetic testing might be suitable for younger patients with a family history of dementia [6].

There are over 100 diseases that can cause dementia, although the four main types include Alzheimer’s disease (AD) (50–75%), vascular dementia (VaD) (15–20%), Lewy body dementia (LBD) (10–15%), and frontotemporal dementia (FTD) (2%). Cognitive impairments, including dementia, are often present in other proteinopathies such as Parkinson’s disease (PD), Huntington’s disease (HD), amyotrophic lateral sclerosis (ALS), and prion diseases [7,8]. Currently, dementia affects more than 55 million people worldwide, and it is estimated that over the next 20–25 years, the number of individuals at risk may exceed 153 million [9]. The prevalence of dementia exhibits an exponential increase, starting at around 1–2% in individuals aged 60–65 years and rising to over 30–35% in people aged over 80 years [10]. It is highly probable that among patients aged 75–80 years, most dementia cases result from a combination of degenerative and vascular factors (mixed dementia). In contrast, cases of pure AD have become less common for people aged 80 and older [10].

AD, the most common type of dementia, is primarily characterized by the accumulation of extracellular amyloid β (Aβ) plaques and intracellular tangles of hyperphosphorylated tau protein in the brain, leading to neural degeneration and synaptic dysfunction. Rare forms of dominantly inherited early-onset AD can result from mutations in the amyloid precursor protein (APP) and presenilin (PSEN1 and PSEN2) genes, collectively accounting for less than 1% of all AD cases [11]. Late-onset AD, which is more prevalent, is typically categorized as sporadic, although there are identified genetic risk factors, with the most important gene coding for apolipoprotein E (APOE) [12]. Beyond the primary risk factors like age, family history, and the *APOE4* genotype [13], late-onset AD is also influenced by additional risk factors, such as triggering receptor expressed on myeloid cells-2 (TREM2), a disintegrin and metalloproteinase 10 (ADAM10), and phospholipase D3 (PLD3), that not only impact APP and tau but also play a role in cholesterol metabolism and immune response [14,15]. In addition, other risk factors associated with AD include environmental and metabolic factors, such as cerebrovascular disease, diabetes, inadequate dietary habits, stress, and head injuries [16]. Progressive decline in memory, particularly episodic memory, as well as problems with executive functions, usually appear in the earlier stage of the disease, whereas challenges related to perceptual motor skills, social cognition, and language abilities tend to become noticeable at a later dementia stage [17]. Additionally, non-memory-related symptoms can also be manifested, including mood disturbances, such as anxiety, depression, and apathy, which may persist throughout the course of the disease [17]. Moreover, during the middle to later stages of dementia, individuals may exhibit behavioral symptoms like aggression, irritability, restlessness, and wandering [18].

VaD, also known as multi-infarct dementia, arteriosclerotic dementia, or vascular cognitive impairment, is the second most common type of dementia resulting from cerebrovascular disease and leading to impaired blood flow to the brain. It can be caused by both large and small vessel diseases, and the critical factor in its development is the location of the lesions rather than the extent of tissue damage [19]. Cerebral autosomal dominant arteriopathy with subcortical infarcts and leukoencephalopathy (CADASIL) is the most prevalent hereditary stroke condition attributed to mutations in the neurogenic locus notch homolog protein 3 (Notch-3) gene and serves as a significant contributor to VaD [20]. It is important to note that risk factors for stroke align with risk factors for VaD, given that stroke represents a significant pathway that connects cardiac and cerebrovascular diseases to vascular brain injury and, ultimately, cognitive impairment. Furthermore, age, diabetes, hypertension, and smoking are some of the other important risk factors for VaD [21]. Although cognitive impairment varies depending on the location and extent of vascular damage, it typically involves deficits in attention, executive function, and processing speed, alongside common symptoms such as alterations in mood and personality [18]. Depression linked to VaD, a condition known as vascular depression, may become apparent in later life, often accompanied by difficulties in executive functions [22].

LBD, the third most common type of dementia, predominantly involves the misfolding and aggregation of α-synuclein, leading to the formation of Lewy bodies, which is a characteristic feature also observed in Parkinson’s disease (PD). This leads to cognitive deficits, which result in impaired attention, executive functions, and visuospatial abilities [17], accompanied by fluctuations in cognitive performance, persistent visual hallucinations, and the presence of parkinsonism [18]. The primary difference between LBD and dementia in PD lies in the chronological order of the occurrence of cognitive and movement symptoms [23]. While in LBD, cognitive impairment occurs before the onset of parkinsonism, in PD, cognitive problems develop after the appearance of motor symptoms [23]. LBD can also manifest with additional features, such as increased sensitivity to specific medications and rapid eye movement (REM) sleep behavior disorder. Clinical indicators that support LBD diagnosis include loss of consciousness, frequent falls, hallucinations, delusions, and depression [24]. The cause of LBD remains elusive, with genetics, age-related changes, and environmental factors playing a role in the etiology; however, further research is needed for its comprehensive understanding [25].

FTD is marked by significant frontal and temporal lobe atrophy, typically containing abnormal tau or ubiquitin protein inclusions. It primarily represents a sporadic condition, although genetics play a significant role in approximately 40% of cases having a familial origin [26] and a quarter of cases showing autosomal dominant inheritance. Key genes implicated in FTD pathogenesis include genes coding for microtubule-associated protein (MAPT), granulin (GRN), and chromosome 9 open reading frame 72 (C9ORF72) [27,28,29]. Additionally, it is assumed that mutations in the *C9ORF72* gene may serve as a link between FTD and amyotrophic lateral sclerosis (ALS), contributing to the incidence of both conditions [30]. Furthermore, thyroid disease and head trauma have been associated with an increased risk of developing FTD [30]. FTD includes clinical subtypes, such as behavioral and language variants, which align with specific regions of brain atrophy. In the behavioral variant, there are notable changes in behavior and personality, encompassing a lack of interest in personal responsibilities, neglect of personal hygiene, isolation from social interactions, and displays of socially inappropriate behavior [31]. Some patients may exhibit repetitive or compulsive motor actions or develop unconventional eating habits [18], leading to potential misdiagnoses, such as major depressive or bipolar disorder. Apart from the behavioral variant, there are three language variants in FTD: the semantic variant, characterized by difficulties in naming and comprehending words; nonfluent aphasia, characterized by the challenges related to speech and/or grammar apraxia; and the logopenic subtype, characterized by issues with word retrieval [32].

The primary obstacles to the effective diagnosis and treatment of dementia revolve around the absence of specific early detection markers and the limited availability of effective therapies. However, recent advancements in genomic medicine have significantly improved our understanding of the underlying causes of dementia. These breakthroughs have led to significant improvements in diagnostic accuracy via the introduction of new biomarkers. Additionally, these advancements made it possible to customize treatment approaches by incorporating pharmacogenetic and pharmacogenomic methods into both drug development and clinical practice [18,29].

## 3. Genetics of Dementia

Genetic factors can play a role in the development of dementia through Mendelian inheritance patterns, leading to high heritability in families, or act as contributing factors in complex heterogeneous multifactorial types of dementia, usually with small effect sizes [33]. As demonstrated in Table 1, Mendelian forms of dementia are usually rare and are characterized by mutations in disease-causing genes, and they are usually inherited through an autosomal dominant pattern [33]. Sporadic forms of different dementias are partly explained by single nucleotide polymorphisms (SNPs), which represent the common type of genetic variation that occurs in a population, and they represent single-letter differences in the DNA sequence at a particular position in the genome and with structural variants (SV), defined as DNA segments of minimum 50 bp, that include duplications, deletions, and insertions of specific genes, as well as their inversions or translocations (Table 1) [34]. Besides genomic variations, epigenetic alternations, such as DNA methylation and hidroxymethylation, histone modifications, non-coding RNA (ncRNA) regulation, and mitochondrial epigenetics, have been included in the pathogenesis of many diseases, including AD [35].

Structural and functional genomics can help identify risk factors associated with dementia and aid in early detection, diagnosis, and drug development. With the development of high-throughput methods for the detection of genetic variants and epigenetic marks on a genome-wide scale, many genes and genomic regions have been implicated in the pathogenesis of AD and other dementias [34,36]. In contrast to candidate-gene association studies, genome-wide association studies (GWAS), whole genome/exome sequencing (WGS/WES), and next-generation sequencing (NGS) provide hypothesis-free approaches to identify novel genes or genomic regions associated with the development or pathology of dementia. These methods lead to the identification of many potential risk variants, which could pinpoint novel biological pathways included in the pathogenesis of dementia [34,36]. However, most variants do not exhibit a direct effect on the protein function; moreover, their individual effect on the total polygenic risk score is usually low, so targeting most of these variants might have little or no therapeutic effects.

Hence, the important step is to implement the functional genomics approaches by integrating the signals obtained by GWAS with other multiomic datasets in order to identify the possible role of these variants and affected biological pathways in the pathogenesis of dementia [37]. The application of CRISPR-Cas gene editing technology significantly enhances the feasibility of large-scale genetic screenings, allowing the usage of precise modifications of the human genome to investigate functional outcomes in human cells, including neurons, microglia, and astrocytes [38]. Integration of functional genomics with genetic studies and single-cell profiling of patient tissues will, therefore, significantly contribute to the uncovering of the complex mechanisms underlying dementia, as well as potential therapeutic targets.

### 3.1. Alzheimer’s Disease

The heritability of neurodegenerative dementias can vary widely between individuals and families, with some genetic overlaps indicating shared biological pathways involved in their development. For example, the overall heritability of AD is estimated to be between 60 and 80%, with significant differences between early-onset AD (EOAD), which develops under the age of 60, represents 5–10% of all AD cases, and has a heritability of 92% to 100% [39], and late-onset AD (LOAD), which develops after 60 years of age, represents the majority of AD cases, is more heterogeneous, and has heritability between 58 and 70% [39,40]. EOAD is mainly caused by mutations in three genes: gene coding for amyloid β precursor protein (*APP*), as well as genes coding for presenilin 1 (*PSEN1*) and presenilin 2 (*PSEN2*), components of γ-secretase, an enzyme involved in the proteolytic cleavage of APP [41]. These mutations, which mostly follow an autosomal dominant pattern, directly result in the overproduction, aggregation, and impaired degradation of Aβ peptides, leading to neurodegeneration; however, they do not show a clear association with LOAD [42]. Tri-allelic polymorphism in the apolipoprotein E gene (*APOE)* was the first identified susceptibility gene for LOAD and has been characterized by missense mutations resulting in the structural and functional differences of the ApoE protein [43]. APOE*4 allele is considered the highest-risk allele with adverse effects on lipid metabolism, cardiovascular diseases, and different proteinopathies, including AD, FTD, LBD, and ALS, while APOE*2 is considered a protective allele [44,45,46]. Besides the prevalent *APOE* polymorphism, which accounts for approximately 25% of genetic variation in AD, rare coding and noncoding alterations within the *APOE* gene have also been associated with the susceptibility to AD [47].

With the development of GWAS, more than 100 additional risk loci for LOAD have been identified, of which 16 lead SNPs are located in the coding exons or in the 3′UTR and 5′UTR, and many others harboring them, as reviewed in [36]. Most of the associated genes are involved in the metabolism of Aβ, in the immune response (especially microglial activation), or in the lipid and endocytosis pathways [48,49,50]. However, most of the identified variants are non-coding and do not have a direct effect on the protein function. It has been suggested that they could act as regulators of gene expression by altering DNA methylation and affecting the binding of transcriptional factors [36]. Although links between SVs and AD were not distinctively found in GWAS [34], many variants showed association with glucuronosyltransferase activity, neuron projection, histone modifications, gene expression, RNA splicing, or protein abundance in post-mortem AD brains, thus providing valuable material for studying their function [51,52,53]. For instance, several GWAS and functional studies identified variants in the *SORL1* gene, coding for sortilin-related receptor 1, which has been involved in the modulation of Aβ peptide production in the brain, to be associated with the risk of LOAD, and possibly familial EOAD [54]. Moreover, SNPs and gene duplication within complement receptor gene 1 (*CR1*), highly expressed in astrocytes and microglia, are considered one of the most important risk variants in AD in several GWAS and are shown to significantly affect the Aβ accumulation in the brain [36,55,56]. In addition, mutations in the *ADAM10* gene, which codes for the component of α-secretase, were shown to attenuate its activity, resulting in the accumulation of Aβ plaques and reactive gliosis in transgenic mice [57]. Significantly altered micro RNA associated with sirtuin 1 (*SIRT1*), β-secretase 1 (*BACE1*), and α-secretase (*ADAM10*) transcripts have also been reported, suggesting potential epigenetic regulation of expression of genes associated with APP metabolism [35].

*BIN1* is the second most important AD susceptibility gene after *APOE*, which encodes for bridging integrator 1 protein, involved in endocytosis, intracellular trafficking, and synaptic plasticity [58,59]. AD-associated *BIN1* variants are non-coding, but they could act as gene expression modulators by facilitating the binding of transcriptional factor MEF2C in primary microglia and induced pluripotent stem cell-derived macrophages [58,59]. Moreover, variants in the triggering receptor expressed on the myeloid cells 2 (*TREM2)* gene have been associated with an increased risk of AD and other neurodegenerative disorders [36]. Functional studies have shown that TREM2 plays a crucial role in regulating microglial activity and that innate immune response may be involved in the Aβ clearance and regulation of tau pathology [60]. Salivary α-amylase AMY1A is an enzyme that degrades polysaccharides such as glycogen and could be responsible for glycogen degradation in astrocytes and neurons that is necessary for neurotransmitter production and memory formation [61]. The high copy number of the *AMY1A* gene possibly leads to higher production of brain α-amylase, which showed an association with lower AD risk and more preserved episodic memory [62]. In addition, a strong association of SNPs, variable number of tandem repeats (VNTRs), and variants generating premature termination codon in the *ABCA7* gene with AD were observed in several GWAS and genetic studies [36]. The *ABCA7* gene codes for the ATP-binding cassette (ABC) transporter involved in lipid homeostasis, cholesterol metabolism, and phagocytosis [63,64]. Several SNPs and retrotransposon Alu insertion into the intron of the *TOMM40* gene [65,66], which is adjacent to and usually in haplotype with *APOE* locus, have also been associated with AD [36]. TOM40 protein is crucial for mitochondrial function, including cell metabolism, apoptosis, and lipid synthesis [67], whereas poly T extension in introne 6 of the *TOMM40* gene is shown to be protective against Aβ toxicity [68]. The targeted analysis demonstrated that duplication of *SULT1A3/4* genes coding for sulfotransferases, which are involved in the metabolism of catecholamines, are associated with the risk of AD and earlier onset of the disease [69]; however, this result was not replicated in the GWAS.

### 3.2. Vascular Dementia

The genetic background of VaD is poorly understood, as it is considered a mostly sporadic disease [70,71]. However, there is supporting evidence that VaD can develop due to single gene mutations, resulting in the development of monogenic disorders, such as CADASIL, which is considered the most common heritable form of VaD [71,72]. CADASIL is caused by mutations in the *NOTCH3* gene, coding for the Notch 3 receptor, which results in impaired function of vascular smooth muscle cells [73]. Much less frequent is cerebral autosomal recessive arteriopathy with subcortical infarcts and leukoencephalopathy (CARASIL), which is developed due to various mutations in the *HTRA1* gene, coding for HTRA1 serine peptidase/protease 1 [74]. Other disorders include Fabry disease (FD), an X-linked lysosomal disease caused by a mutation of the *GLA* gene, resulting in impaired α-galactosidase activity and accumulation of glycosphingolipids [75]; retinal vasculopathy with cerebral leukodystrophy (RVCL) due to frame-shift *TREX1* mutations that result in a DNase III exonuclease impairment [76]; cerebral amyloid angiopathy (CAA), characterized by defective protein deposits, including Aβ and highly affected by mutations in *APP*, *PSEN1,* and *PSEN2* genes but also in transthyretin (*TTR*), cystatin C (*CST3*), gelsolin (*GSN*), and integral membrane protein 2B (*ITM2B*) genes [77]; and disorders related to mutations in collagen type IV α1 chain (*COL4A1*) gene, such as small vessel arteriopathy and cerebral small vessel disease (CSVD) [78].

The genetic basis of VaD sporadic forms is mostly based on candidate-gene studies, and it overlaps with the genetic background of the vascular risk factors such as hypertension, dyslipidemia, and smoking, as well as of other diseases, such as AD and stroke [71,72]. APOE*E4 allele was associated with a higher risk of VaD in several meta-analyses, irrespective of patient ethnicity [71,79]. Genetic variants in the methylenetetrahydrofolate reductase (*MTHFR*) gene, which affects the level of homocysteine [79]; polymorphisms in the paraoxonase 1 (*PON1*) gene [80]; insertion–deletion variant in the intron 16 of *ACE* gene, coding for the angiotensin-converting enzyme, associated with vascular reactivity, have also been implicated in the moderating the risk of development of sporadic VaD [81]. SNPs in the genes related to the inflammation, such as interleukin (*IL-1α*, *IL-1β*, *IL-6*), and tumour necrosis factor *(TNF-α*, *TGF-β1)* genes, could also possibly influence VaD development; however, these findings were not replicated in all ethnic groups [72]. GWAS also detected associations of VaD with polymorphisms in the androgen receptor (*AR*) gene on the X-chromosome and *RPGRIP1L* gene, whose product regulates thromboxane A2 and consequently vasoconstriction and platelet aggregation [72,82], while functional studies confirmed the association of spleen associated tyrosine kinase (*SYK*) and pleckstrin homology like domain family B member 2 (*PHLDB2*) genes with VaD [83,84]. The challenge in determining the genetic basis of sporadic VaD is due to the small effects of many genetic variants, as well as the heterogeneity of VaD phenotypes. Therefore, it is necessary to confirm these findings in large replication cohorts and to further explore the biological mechanisms involved in both AD and stroke [72].

### 3.3. Frontotemporal Dementia

The prevalence of familial FTD represents 30% of the total FTD cases [85]. It develops mostly due to autosomal dominant mutations in chromosome 9 open reading frame 72 (*C9ORF72*), microtubule-associated protein tau (*MAPT)*, and progranulin *(GRN)* genes, which are responsible for 60% of familial FTD cases [33,86]. The sporadic form, which represents 70% of FTD cases, is more complex, and its heritability ranges from 26 to 31% and mostly includes SNPs [85]. Pathogenic expansion of GGGGCC hexanucleotide repeats in the intron region of the *C9ORF72* gene is the most common genetic cause of FTD and ALS and a rare cause of PD. It accounts for the 20–30% genetic susceptibility of familial and about 6% of sporadic FTD [87,88]. The exact function of the protein encoded by the *C9ORF72* gene is not well known, but it appears to be involved in the regulation of autophagy and inflammation [89]. Mutations in the *C9ORF72* gene can lead to both loss-of-function and gain-of-function effects by forming RNA foci in the nucleus, which can be translated into dipeptide repeat proteins and TAR DNA binding protein (TDP-43) inclusions in neurons and oligodendrocytes [89]. Complex inversion of the 673 bp region in the *MAPT* gene (H2 haplotype) has been associated with FTD/ALS but also with AD and LBD risk. Mutations in exonic and intronic regions of the *MAPT* gene primarily affect the mRNA splicing, which can lead to disruption of the tau protein structure, resulting in impaired microtubule assembly and aggregation of tau filaments [90]. In addition, complex inversion 673 bp region of *MAPT* H2 haplotype can reduce the risk of FTD/ALS but also AD, LBD, and PD [34,91,92], while several identified deleterious SVs encompassing the *MAPT* gene region and H1/H2 haplotype could be implicated in the gene expression [34,51]. *GRN* gene mutations are mostly non-sense and deleterious mutations, which generate a premature termination codon that leads to reduced expression of progranulin [93] and, consequently, in lysosomal impairment and accumulation of pathological forms of ubiquitinated TDP-43, characteristic for some types of FTD and ALS [94,95].

In addition, more rare mutations were associated with FTD with cumulative risk <5%, of which the strongest effect was loss-of-function mutations in tank-binding kinase (*TBK1)* gene, coding for serine/threonine kinase, which are estimated as the fourth and second most common genetic cause of FTD and ALS, respectively [96]. *TBK1* mutations result in a dysfunctional vesicular transport system, which could lead to deregulated autophagy and neurodegeneration [97]. Other associated genes are mostly involved in the regulation of transcription and RNA splicing, protein degradation, membrane fusion, autophagy, and apoptosis [98] and include genes coding for valosin-containing protein (*VCP*), optineurin (*OPTN*), TAR DNA binding protein (*TARDP*), charged multivesicular body protein 2B (*CHMP2B*), triggering receptor expressed on myeloid cells 2 (*TREM2*), ubiquilin 2 (*UBQLN2*), sequestosome 1 (*SQSTM1*), fused in sarcoma (*FUS*), coiled-coil-helix-coiled-coil-helix domain containing 10 (*CHCHD10)*, sigma non-opioid intracellular receptor 1 *(SIGMAR1)*, cyclin F (*CCNF),* and TIA1 cytotoxic granule associated RNA binding protein (*TIA1)* [85]. Additional high-risk loci containing common genetic variants (SNPs) were identified and replicated in the recent study [99], such as several variants located in the introns of *LOC730100* gene, coding for long ncRNA, which upregulation has been shown to enhance proliferation and invasion of glioma cells [100]; *CEP131* gene, coding for centrosomal complex involved in the stabilization of genome [101]; *ENTHD gene 2*, involved in the trans-Golgi network vesicular processes [102]; and *C17orf89* gene [99].

### 3.4. Lewy Body Dementia

The majority of LBD are sporadic cases (>80%), and genetic influence on its development was previously considered small; however, it is now clear that the genetic component of LBD is estimated to be 36–59.9%, based on SNPs only [103,104]. Moreover, there is increasing evidence of hereditary components in the development of LBD, which is also found in related dementia, such as AD- and PD-associated dementia [103,104]. Not only does LBD share similar clinical and neuropathological features with PD and in a subset of AD cases, but also similar genetic factors have been implicated in the development of these diseases, suggesting similar molecular pathways underlying their pathogenesis [103,105]. However, recent findings have shown genetic variants specific to LBD [106]. Well-established risk genes for LBD include the *APOE* gene, also associated with AD, as well as α-synuclein (*SNCA*) and β-glucosylceramidase (*GBA*), which also represent risk genes for PD [103,104]. *APOE* risk alleles have been implicated in the pathology of AD [107] and LBD but not PD [108], which could explain the presence of AD-related neuropathological hallmarks in numerous LBD cases [109]. Point mutations in the *SNCA* gene are possibly affecting the membrane binding activity and synuclein aggregation, while locus multiplications of *SNCA*, leading to the overproduction of synuclein, can result in the formation of Lewy bodies [110]. Besides potential disease-causing mutations, there are several SNPs in the *SNCA* locus that could modulate the risk of developing LBD and PD, with differential prevalence between these diseases [106]. Moreover, *SNCA* gene methylation was suggested to be significantly decreased in LBD, leading to higher gene expression [111]. Mutations in the *GBA* gene, which codes for lysosomal enzyme β-glucocerebrosidase, lead to reduced enzyme activity, resulting in impaired degradation of α-synuclein and its accumulation [112], and are linked with the higher risk of PD, with variations associated with earlier onset and shorter life-span in PD and LBD [113].

The latest GWAS identified 13 genomic risk loci significantly associated with LBD, contributing to 6.24% of total LBD heritability [105,106]. They include variations in *BIN1* gene (also associated with AD); transmembrane protein 175 and lysosomal K^+^ channel *TMEM175* gene (implicated in PD), which deficiency leads to decreased lysosomal catalytic activity due to pH imbalance [114]; *CLU* gene, coding for clusterin, a protein that possibly binds α-synuclei aggregated species [115]; *FBXL19* gene, which encodes for the type of ubiquitin ligases involved in the regulation of ubiquitination and degradation of inflammatory cytokines with potential neuroprotective effect [116]; and the *MAPT* gene, which is also involved in the pathogenesis of FTD and AD [117,118]. Functional enrichment analysis showed that many variants associated with LBD were found in regions associated with the regulation of gene transcription and translation, such as exone regions, enhancers, and regions linked to histone modifications, especially H3K36me3 [105]. A common structural variant (309 bp deletion) in the intron region of the two-pore calcium channel (*TPCN1)* gene that encodes a two-pore calcium channel has been associated with the risk of LBD and AD [119]. The functional implications of this gene were confirmed in *Tpcn1* knockout mice, who have shown impaired memory and spatial learning [120]. Moreover, deletion in the *OPTN* gene was associated with an increased risk for LBD [119]. Accumulation of optineurin in Lewy bodies [121] and previous involvement of *OPTN* mutation in the development of FTD [122] confirm the importance of this gene in the pathogenesis of neurodegenerative dementias. These results showing genetic overlap and potentially shared biological mechanisms involved in AD, FTD, PD, and LBD could provide insight into both the prevention and treatment of these diseases.

## 4. Therapeutic Strategies in Dementia

One of the primary goals in treating various forms of dementia is to decrease cognitive, behavioral, and psychological symptoms while also attempting to slow the progression of the disease. Pharmacotherapy is frequently one of the initial strategies employed to address symptoms or hinder the progression of disease, with a primary focus on targeting the impairment of cholinergic and glutamatergic systems [123]. At present, the Food and Drug Administration (FDA) has approved two classes of pharmacological medications for managing the cognitive symptoms of AD: acetylcholinesterase (AChE) inhibitors and N-Methyl-D-Aspartate (NMDA) receptor antagonists. However, these medications are not effective in slowing down the progression of the disease itself but only provide relief from cognitive symptoms without altering the course of the underlying disease [123,124].

Both AChE-selective inhibitors, donepezil and galantamine, and dual AChE and butyrylcholinesterase (BuChE) inhibitor, rivastigmine, promote the increase in AChE levels in the synaptic cleft [6]. AChEIs prevent the breakdown of acetylcholine by inhibiting the action of acetylcholinesterase, leading to an increase in cholinergic neurotransmission [125]. Donepezil, rivastigmine, and galantamine are currently approved for treating mild to moderate symptoms of AD and have shown modest positive effects on cognitive symptoms [2]. Since their introduction into clinical practice, these drugs have remained the standard approach to the symptomatic treatment of AD. Various systemic reviews concluded that AChEI treatment of dementia patients shows small but significant improvement in cognitive function [126,127]. A slow dose titration of these drugs is recommended to reach the optimal dose with minimal adverse effects [6]. Even with a gradual titration process, these medications can still lead to gastrointestinal and neurological issues, including symptoms like nausea, vomiting, diarrhea, abdominal pain, dizziness, weight loss, tremor, and fatigue [6]. In such cases, the medication dosage may need to be reduced, or an alternative drug can be considered [128]. In addition to AD, cholinergic deficiencies are also observed in other forms of dementia, like dementia associated with PD and LBD [129,130]. While AChEIs are not officially approved for these dementia types, there is growing evidence supporting their use in alleviating neuropsychiatric symptoms of patients diagnosed with LBD and PD [128]. However, these drugs failed to show benefits among individuals with MCI [131].

Memantine is an NMDA receptor antagonist that reduces the impact of glutamate-induced excitotoxicity [132]. It prevents the over-activation of glutamate receptors by slowing down the flow through the NMDA-receptor subtype of glutamate receptors [133]. This way, memantine prevents the overactivation of the glutamatergic system, still maintaining its normal function. It is used as monotherapy to manage symptoms of moderate and severe AD. Additionally, research has demonstrated its beneficial effects in slowing down the progression of cognitive decline in individuals with AD [134,135]. Memantine treatment of patients with VaD showed minimal improvement in cognitive status [136,137]; however, in patients with LBD, there were no significant effects on cognitive or behavioral symptoms [138,139]. In addition, when AChEI is no longer effective, memantine is an alternative drug for patients with moderate and severe AD [128]. Memantine is usually better tolerated than AChEI, but in some cases, it can cause headache, fatigue, and gastric pain [128]. Moreover, the combination of donepezil and memantine has been well tolerated, with positive effects on cognition and performing daily activities [140].

Anti-amyloid drug therapy based on monoclonal antibodies is one of the novel approaches for slowing down the progression of AD [141]. Aducanumab is a monoclonal antibody reported to be effective in identifying Aβ aggregates and selectively binding to both oligomeric and fibrillary states rather than amyloid monomers [142]. It was reported that aducanumab has beneficial effects in reducing Aβ plaques in patients with mild AD or MCI and was approved in 2021 by the FDA [143]. However, its approval was met with controversy due to mixed results in clinical trials, with some experts questioning the drug’s efficacy and long-term benefits [144,145,146]. Specifically, although some patients experienced a reduction in Aβ levels, it remains uncertain whether these results have a clinical impact on cognitive functions [144]. Another monoclonal antibody, lecanemab, received approval from the FDA in 2023 for the treatment of people with MCI or mild dementia due to AD who have elevated Aβ levels in the brain. Various clinical trials suggested that lecanemab reduces Aβ in the brain [147,148,149]. Other drugs targeting Aβ plaques, such as donanemab, are currently in clinical trials [150]. However, the full extent of clinical efficacy, long-term benefits, and safety of these drugs is yet to be investigated.

Due to the high complexity of neurodegenerative diseases, single-target therapy approaches have been mainly ineffective in preventing or slowing the progression of these diseases. Additionally, a significant challenge lies in the occurrence of adverse effects and the development of drug tolerance [124,151]. As a result, multi-target strategies are increasingly being considered, particularly in the case of AD, and a great number of structures based on this polypharmacology concept have been proposed [152]. The main focus of this approach involves the design of a single ligand with pleiotropic effects capable of simultaneously interacting with at least two therapeutic targets. There are three types of polypharmacological ligands, which are classified as conjugate, fused, and merged ligands [153]. Conjugate ligands are composed of pharmacophoric structures linked by a stable or cleavable molecule, allowing them to be released and interact with multiple targets. Fused ligands have pharmacophoric structures that are joined but do not overlap, whereas merged ligands have extensive overlap in their pharmacophoric structures, resulting in smaller and more straightforward molecules [153].

Over the past decades, multi-target therapeutic compounds targeting cholinesterase inhibition, anti-inflammatory and antiapoptotic activity, monoamine oxidase (MAO) inhibition, and neuroprotection have been investigated [154], particularly focusing on AChE [155], BuChE [156], β-secretase 1 (BACE-1) [157], cannabinoid receptor subtype 2 (CB_2_R) [158], serotonin (5-HT) receptors [159], serotonin transporter (SERT) [160], cyclooxygenase-2 (COX-2) [161], 5-lipoxygenase (5-LOX) [162], and nuclear factor erythroid 2-related factor 2 (Nrf2) [163]. The majority of multi-target compounds currently undergoing investigation for AD treatment are specifically designed to moderate cholinesterase and monoamine activity; inhibit Aβ aggregation; and exert metal-chelating, anti-neuroinflammatory and antioxidant activity [164]. The development of novel drugs is inspired by established and approved medications, like donepezil [165,166] and rivastigmine [167], as well as by various natural bioactive derivatives, such as resveratrol, flavonoids, or curcumin [168,169]. An example of a multi-targeted drug candidate is ladostigil, which functions as both AChEI and a brain-selective inhibitor of MAO-A and MAO-B [34]. It is primarily intended for the treatment of dementia, especially AD, PD, and depression [35,36]. This compound is developed by combining the carbamate rivastigmine with the N-propargyl scaffold from an anti-parkinsonian drug and the irreversible selective MAO-B inhibitor, rasagiline [35]. It was demonstrated that ladostigil is safe and well-tolerated, but it did not show significant effectiveness in delaying the progression of dementia. However, it did display the potential to reduce the brain and hippocampus volume loss, indicating a possible impact on atrophy [37]. Despite encouraging preclinical results, so far, no multi-targeted drug has received approval for dementia treatment. However, as research into the underlying mechanisms of the disease continues, and advances in multi-target drug discovery for AD unfold, multi-targeted ligands hold substantial promise as a potential pharmacotherapeutic strategy for dementia.

Numerous research studies have reported a wide range of non-cognitive symptoms in dementia patients, including behaviors such as aggression, agitation, and psychosis, as well as issues related to eating and mood disorders [170]. Collectively, these non-cognitive symptoms are referred to as behavioral and psychological symptoms of dementia (BPSD) [171]. In addition to anti-dementia drugs, pharmacological treatment of BPSD comprises antidepressants, antipsychotics, benzodiazepines, and mood stabilizers [172,173,174,175]. While tricyclic antidepressants and paroxetine are not recommended due to certain anticholinergic side effects, selective serotonin reuptake inhibitors (SSRIs), such as sertraline and citalopram, as well as tradozone, have shown good tolerability and effects in reducing agitation, tension, aggression, psychosis and sleep disturbances [174,175]. Due to adverse effects, the administration of antipsychotics and mood stabilizers is also not recommended for BPSD therapy, with the exception of atypical antipsychotics risperidone, olanzapine, and aripiprazole, as well as valproic acid [172,173]. More recently, FDA-approved two drugs for treatment of BPSD: suvorexant, an orexin receptor antagonist, approved in 2020 for the treatment of insomnia in individuals with mild to moderate AD, and brexpiprazole, atypical antipsychotic, approved in 2023 for the treatment of agitation associated with AD.

Aside from pharmacological treatments, there is a recommendation to consider non-pharmacological approaches for BPSD treatment, as well as to increase the quality of life for both patients and their caregivers [176]. The aim of non-pharmacological interventions is to enhance or, at the very least, maintain cognitive function, enabling individuals to carry out their regular daily activities while effectively managing the behavioral symptoms associated with cognitive impairment. Non-pharmacological interventions include various disciplines, each of them attempting to have a positive effect on cognition, mood, and other behavioral and psychological symptoms of dementia [177]. Several non-pharmacological treatments have been proposed for targeting cognitive functional aspects of people with dementia. Sensory and multi-sensory stimulation includes visual, olfactory, tactile, taste, and kinaesthetic stimulation in order to reduce agitation and increase awareness [178]. These types of stimulation include art therapy, aromatherapy, light therapy, music, and dance therapy, as well as snoezelen multi-sensory therapy. Cognitive and emotion-oriented care intervention is useful for improving cognitive, emotional, and social functioning [179]. Commonly used treatments include reminiscence therapy, reality orientation therapy, and validation therapy [180]. There is also behavioral management therapy that has been reported effective in suppressing or eliminating stereotypical behavior, such as wandering and incontinence [181,182]. Other therapies have been applied, such as animal-assisted therapy, home adaptation therapy, and assistive technologies [180]. These types of interventions have been found to be useful in improving outcomes and quality of life in patients with dementia [183].

Non-pharmacological techniques have been reported to be more effective with fewer side effects when compared to pharmacotherapy with antipsychotics [176,184,185]. There are several proposed recommendations for reducing responsive behavior, including apathy, hyperactivity, and psychosis [186], maintaining or improving functional capacity, and reducing comorbid emotional disorders, such as anxiety and/or depression [187]. These symptoms are frequently observed in individuals with dementia, and while medication therapy may be necessary, it is generally recommended that non-pharmacological interventions are used as the primary approach. [188]. Sensory stimulation, such as music and light therapy and validation therapy, has been effective in reducing these types of behavior [187]. Interventions for improving functional capacity, which refers to cognitive function and improving well-being and daily life activity, should include cognitive stimulation, reminiscence for cognitive function, as well as exercise and light therapy for improving daily life activities [189,190]. Furthermore, exercise, music therapy, reminiscence, validation therapy, and psychological treatments should also be applied to reduce symptoms of depression and anxiety [191,192].

Therefore, it is important that non-pharmacological treatments become an integral part of the management of dementia symptoms and rehabilitation programs [181]. The field of dementia care continually evolves, with new therapies regularly joining the available options for managing this condition. However, it is essential to recognize that no single method alone provides a comprehensive long-term solution for dementia management [181] and that complementary approaches are needed in order to enhance the long-term care and quality of life for individuals with dementia.

## 5. Pharmacogenomics of Cognitive Symptoms: Conventional Anti-Dementia Drugs

When we talk about pharmacogenomics studies related to the treatment of cognitive symptoms in dementia, most of them are focused on AChEIs, memantine, and combined treatments with these four medications (Table 2). The AChEIs and memantine have different metabolic pathways. Both donepezil and galantamine are metabolized mostly by CYP3A4, CYP2D6, and CYP1A2 enzymes in the liver, while rivastigmine undergoes cholinesterase-mediated hydrolysis and its metabolism minimally relies on major cytochrome P450 isozymes [193]. Memantine is metabolized to a minor extent by the liver and is mainly excreted unchanged by the kidneys [194]. Around 15–20% of patients diagnosed with AD exhibit aberrant AChEI metabolism, with approximately half of them being ultra-rapid metabolizers and the other half slow metabolizers [195].

Donepezil is the most prescribed drug for the treatment of cognitive symptoms in dementia [195]. Different *CYP2D6* variants have been studied in order to assess their influence on donepezil efficacy and safety in AD patients [195,196,197,198,199,200,201,202,203,204]. These variants include rs1065852, rs1080985, CYP2D6*3 (rs35742686, 2549delA), CYP2D6*4 (rs3892097, 1846G>A), CYP2D6*6 (rs5030655, 1707delT), CYP3A4*1B (rs2740574, −392A>G), and CYP2D6*10 (rs1065852, 100C>T); however, the results are inconsistent [203]. *CYP2D6* rs1080985 (−1584C/G) is one of the most studied polymorphisms in the context of its association with the clinical efficiency of donepezil. The rs1080985 G allele defines the CYP2D6*2A variant, which was found to be potentially associated with a higher drug metabolism rate [197,205]. CYP2D6 poor metabolizers were found to have a 32% slower clearance rate and a 67% slower metabolism rate of donepezil compared to ultra-rapid metabolizers [202].

Polymorphism rs1065852 (100C>T) appears in CYP2D6*4 and CYP2D6*10 variant. The study in Han Chinese patients with AD found that the CYP2D6*10/*10 allele was associated with better efficacy and higher steady-state plasma concentration of donepezil compared to other *CYP2D6* genotypes [206,207]. The efficiency of donepezil has also been associated with its interaction with CYP3A4/5 [208]. A study by Noetzli and colleagues analyzed the effect of different *CYP3A* gene variants on donepezil clearance in AD patients. They studied CYP3A4*1B (rs2740574), *CYP3A4* (rs4646437), CYP3A4*22 (rs35599367), CYP3A5*3 (rs776746), and CYP3A7*1C (−262T > A and −270T > G) variants and found no connection with donepezil pharmacokinetic parameters [202]. A similar result was reported by Magliulo and colleagues in Italian subjects diagnosed with AD. The study investigated CYP3A4*1B, CYP3A4*3 (rs4986910), CYP3A4*4, CYP3A5*2 (rs28365083), CYP3A5*3 (rs776746), and CYP3A5*6 (rs10264272) and found no association between these variants and donepezil concentration in plasma samples [209]. The lack of influence of *CYP3A4* variants on donepezil efficiency was also reported in Chinese AD patients [210].

The efficiency of donepezil could also be influenced by other genetic factors that are not directly involved in its metabolism (Table 2). Some of the potential candidates are genes coding for apolipoprotein E (*APOE*), ATP-binding cassette (ABC) transporter (*ABCA1* and *ABCB1*), butyrylcholinesterase (*BCHE*), acetylcholine receptor subunit α7 (*CHRNA7*), choline acetyltransferase (*ChAT*), estrogen receptor gene (*ESR1*), or paraoxonase (*PON-1*). Apolipoprotein E is known to be involved in lipoprotein metabolism and associated with a higher risk of developing AD [211]. Several studies have suggested its association with the efficacy of donepezil treatment. Patients with AD, carriers of the high-risk *APOE* ε4 allele, were found to have a better response to donepezil treatment and more significant improvement of cognitive symptoms [212,213]. However, there are also studies reporting opposite results [214] or no association between *APOE* and treatment efficiency of donepezil [206,207,215,216]. The study by Lu and colleagues suggested that the *APO ε3* allele could moderate the efficiency of donepezil treatment by demonstrating better treatment response in subjects who were not *APOE* ε3 allele carriers [217]. Moreover, it seems that combined *APOE* and *CYP2D6* influence on donepezil treatment efficacy might be explained by their involvement in lipid metabolism and liver function [217,218].

Two ABC transporters have also been suggested as possible modulators of donepezil efficacy, ABCB1 and ABCA1. The results of different studies reported no association between the efficacy of donepezil and different *ABCB1* polymorphisms [202,207,209]. Another interesting genetic factor in donepezil pharmacogenetics is the cholesterol transporter ABCA1. Its function is to moderate Aβ aggregation and stimulate the clearance of Aβ peptides [203]. The study by Lu and colleagues suggested that patients who were *ABCA1* rs2230806 GG genotype carriers had better responses to treatment with donepezil than AA and GA genotype carriers. The combined effect between *APOE* and *ABCA1* genetic variants was also suggested, indicating that patients who were *APOE ε3* non-carriers and *ABCA1* rs2230806 GG homozygotes responded better to donepezil [219].

Evidence supporting the role of estrogen in cognitive function has raised the question of potential association between *ESR1* gene variants and the therapeutic effects of AChEIs [220]. Two *ESR1* polymorphisms, rs2234693 and rs9340799, were examined in AD patients receiving donepezil, rivastigmine, or no treatment [221]. The authors observed a significant effect of *ESR1* variants in patients treated with donepezil and reported better treatment response in women than in men [221]. The BCHE is a member of the cholinergic enzyme family. It is mainly synthesized in the liver; however, it is also present in the central and peripheral nervous system [222]. The most researched polymorphism of *BCHE* is rs1803274, also known as the K-variant, which has been associated with up to 7% reduction in enzyme hydrolytic activity in heterozygotes (AG) and 14% reduction in homozygotes (AA) [223]. This polymorphism has been associated with poor treatment response in patients receiving donepezil [224]. However, two other studies did not confirm this association, showing no significant relationship between the presence of K-variant [225,226] or rs1355534 [226] polymorphism and donepezil efficacy. A study by De Beaumont and colleagues demonstrated that AD patients, who are carriers of *APOE* ε4 and *BCHE* K-variant, have an earlier age of onset, accelerated cognitive decline and better response to donepezil therapy [227].

*The chAT* gene encodes an enzyme, choline acetyltransferase, responsible for the biosynthesis of acetylcholine. Two genetic variants of the *ChAT* gene have been associated with response to AChEI treatment, rs2177370 and rs3793790 [228]. These polymorphisms have been associated with impaired synthesis of acetylcholine. Results suggest that the CC haplotype is responsible for the decreased synthesis of acetylcholine, while carriers of the CT haplotype demonstrated a higher acetylcholine synthesis rate [228]. The association of rs2177370 polymorphism with AChEI efficacy was also reported by Harold and colleagues [229], while other studies did not observe such a connection [226]. Lee and colleagues analyzed the difference in donepezil treatment response between carriers and non-carriers of the rs3810950 (2384G>A) A allele and found that the treatment outcome, after 26 weeks of therapy, is positively influenced by the presence of A allele [230].

Another potential candidate in the pharmacogenetics of donepezil is the *CHRNA7* gene, which encodes the α7 subunit of the nicotinic acetylcholine receptor (nAChR). Polymorphisms in *CHRNA7* could affect the binding of acetylcholine, which is increased due to donepezil treatment, to nAChRs. A longitudinal study in the Brazilian population demonstrated a significant association between *CHRNA7* rs6494223 polymorphism (T allele) and the efficacy of donepezil [231]. The association was present after 6 months of treatment; however, after 2 years of follow-up, the association could no longer be detected [231]. Another study found an association between *CHRNA7* rs8024987 (C→G) polymorphism and the outcome of AChEI therapy, but only in female patients [232]. The same SNP was investigated by Clarelli and colleagues, but the results did not confirm the finding reported by Weng et al. [233]. Two SNPs, rs885071 (T→G) and rs8024987 (C→G), were found to be in linkage disequilibrium and associated with treatment response [232].

Arylesterase PON-1 has an important role in protecting cells from injuries caused by oxidative stress. Reduced PON-1 serum levels and activity have been associated with AD [234]. The most studied *PON-1* polymorphism is rs662 (Q192R, A>G), glutamine to arginine substitution at amino acid residue 192 [235]. Pola and colleagues were able to associate this polymorphism with AChI treatment (donepezil and rivastigmine) response, showing a higher frequency of the R allele, which exhibits higher enzyme activity, in patients who had good response to therapy [236]. Since PON-1 acts as an endogenous cholinesterase inhibitor, it is possible that it synergistically interacts with other AChEIs and improves their efficacy [236].

As in the case of treatment response to donepezil, variability in rivastigmine efficiency could be explained by the effect of different gene variants (Table 2). Some of the potential candidates are *APOE*, *BCHE*, presenilin (*PSEN*), and UDP glucuronosyltransferase 2B7 (*UGT2B7*) genes. Better efficacy of combined rivastigmine and memantine therapy has been reported in *APOE* ε4 carriers [237]. A multicenter study by Blesa and colleagues reported no association between *APOE* ε4 allele and the response to treatment with rivastigmine [238]. The retrospective analysis by Farlow et al. investigated the efficacy of rivastigmine on cognitive performance in AD patients, taking into consideration the *APOE* genotype. The study reported more pronounced symptom improvement in subjects who were not *APOE* ε4 carriers in both rivastigmine and placebo groups [239]. Similar to donepezil treatment efficacy, the BCHE K-variant affects the response to rivastigmine treatment, especially in the presence of the *APOE* ε4 allele [240].

Presenilin is a subunit of γ-secretase, an enzyme that is crucial in processing APP, thus producing small peptides, including Aβ. Different mutations in the *PSEN2* gene can lead to increased production of Aβ, including a common single adenine (A) nucleotide deletion polymorphism, which is located in the upstream promoter region of this gene [241]. Zamani and colleagues reported the best treatment response to rivastigmine in AD patients with PSEN2 +A/−A genotype, alone or in combination with *APOE* ε3/ε3 or *APOE* ε4/ε4 genotype, while individuals with combined *PSEN2* +A/+A and *APOE* ε3/ε4 genotypes had the worst response to treatment [242]. UDP glucuronosyltransferase 2B7 is a metabolic enzyme important in the elimination of endogenous compounds and potentially toxic xenobiotics [243]. Different polymorphisms in the *UGT2B7* gene could alter the enzyme activity and, thus, affect the biotransformation of its substrates [244]. The study by Sonali and colleagues investigated the effect of *UGT2B7* (802C>T, UGT2B7*2, rs7439366) polymorphism on rivastigmine efficiency, alone and in combination with memantine [245]. Results suggested that carriers of the *UGT2B7* variant, who were poor metabolizers, had poor clinical response to rivastigmine therapy [245]. However, the study had a limited sample size, and further research is necessary to confirm or dispute these results.

As already mentioned, galantamine is metabolized mainly by CYP3A4 and CYP2D6 enzymes, which is why *CYP2D6* genetic variants have been associated with the outcome and side effects of galantamine treatment (Table 2). A study by Ma and colleagues detected better treatment response in AD patients who were CYP2D6*10 rs1065852 carriers and reported fewer adverse side effects [210]. Genetic variants of *CHRNA7* are also interesting targets in pharmacogenetic studies focused on galantamine efficacy. Better treatment response to galantamine was reported in patients carrying minor allele variants of rs8024987 (C/G) or rs6494223 (C/T) polymorphism [246].

Unlike in the case of AChEIs, there are not many studies that focus on the pharmacogenetics of memantine efficacy (Table 2). From in vitro studies, we know that cytochrome P450 isozymes are not involved in the metabolism of memantine. Memantine is a substrate of the human organic cation transporter 2 (OCT2) [247], but its clearance is probably also related to other transporters, including organic cation/carnitine transporters (OCTN 1-3), the multidrug and toxin extrusion proteins (MATE1-2), and P-glycoprotein (P-gp) [247,248]. Some studies also suggest the involvement of nuclear receptors in the regulation of cation transporters, including pregnane X receptor (PXR), constitutive androstane receptor (CAR), and peroxisome proliferator-activated receptor (PPAR) [249,250,251]. Genetic variations in different membrane transporters could be associated with variability in memantine pharmacokinetics [248]. Pregnane X regulates the expression of metabolic enzymes and transporters, which are involved in drug metabolism [252]. The polymorphism rs1523130, located in the *NR1I2* gene, which encodes pregnane X, was shown to modulate memantine elimination [248]. Memantine clearance was found to be 16% slower in patients carrying at least one T allele (CT and TT genotype) [248]. Ovejero-Benito and colleagues investigated the association of 67 polymorphisms in 21 genes, including *CYP2D6*, *CYP2C9*, *CYP2A6*, *ABCB1*, and genes coding for different neurotransmitter receptors, with donepezil or memantine pharmacokinetics and safety. The authors reported no significant association of analyzed SNPs with both memantine and donepezil pharmacokinetics or adverse drug reactions [196].

**Table 2:** Pharmacogenomics of conventional anti-dementia drugs.

Drug Name	Drug Class	Associated Gene	Pharmacogenetics Finding	References
Donepezil	Acetylcholin- esterase inhibitor	*CYP2D6*	Functional alleles (rs1080985, rs1065852) affect variability in donepezil efficacy	[197,202,205,206,207]
		*CYP3A4/5*	Lack of association with donepezil pharmacokinetic parameters	[202,209,210]
		*APOE*	Carriers of high-risk *APOE* ε4 allele have better response to donepezil treatment	[212,213]
			No association between *APOE* and treatment efficacy	[206,207,215,216]
			Better treatment response in *APOE* ε3 allele carriers	[217]
			Combined *APOE* and *CYP2D6* influence on donepezil treatment efficacy	[217,218]
		*ABCB1*	No association between efficacy of donepezil and *ABCB1* polymorphisms	[202,207,209]
		*ABCA1*	*ABCA1* rs2230806 influences donepezil treatment response. Combined effect of *APOE* and *ABCA1* genetic variants	[219]
		*ESR1*	Effect of *ESR1* variants (rs2234693, rs9340799) in donepezil-treated patients	[221]
		*BCHE*	*BCHE* rs1803274 (K-variant) is associated with donepezil poor treatment response	[224]
			No relationship between K-variant or rs1355534 polymorphism and donepezil efficacy	[225,226]
			Carriers of *APOE* ε4 and *BCHE* K-variant have better response to donepezil therapy	[227]
		*ChAT*	Polymorphisms rs2177370, rs3793790 and rs3810950 associated with AChEI efficacy	[228,229,230]
			No association with treatment response	[226]
		*CHRNA7*	Association between *CHRNA7* variants (rs6494223, rs8024987, rs885071) and donepezil efficacy	[231,232]
			No association between rs8024987 and treatment response	[233]
		*PON-1*	*PON-1* rs662 associated with AChI treatment (donepezil and rivastigmine)	[236]
Rivastigmine	Acetylcholin- esterase inhibitor	*APOE*	Better efficacy of combined rivastigmine and memantine therapy in *APOE* ε4 carriers	[237]
			No association between *APOE* ε4 allele and treatment response	[238]
			Better improvement in non-carriers of *APOE* ε4 in rivastigmine and placebo group	[239]
		*BCHE*	*BCHE* K-variant affects treatment response, especially in presence of *APOE* ε4 allele	[240]
		*PSEN2*	Best treatment response in patients with *PSEN2* +A/−A genotype, alone or in combination with *APOE* ε3/ε3 or *APOE* ε4/ε4 genotype	[242]
		*UGT2B7*	Poor metabolizers with *UGT2B7* variant (UGT2B7*2, rs7439366) had poor clinical response	[245]
Galantamine	Acetylcholin- esterase inhibitor	*CYP2D6*	Better treatment response and fewer adverse effects in CYP2D6*10 rs1065852 carriers	[210]
		*CHRNA7*	Better treatment response in carriers of minor allele variants of rs8024987 or rs6494223 polymorphism	[246]
Memantine	NMDA receptor antagonist	*NR1I2*	Memantine clearance was 16% slower in carriers of at least one rs1523130 T allele (CT and TT genotype)	[248]
		*CYP2D6*	No significant association with memantine pharmacokinetics or adverse drug reactions	[196]
		*CYP2C9*		
		*CYP2A6*		
		*ABCB1*		

## 6. Pharmacogenomics of Cognitive Symptoms: Multifactorial Treatments

In the early 2000s, more extensive research began on the effect of a combination of drugs on patients with different variations of genes essential for the onset of dementia. The effects of multifactorial therapy based on pharmacogenomics are most thoroughly described through the therapeutic response related to *APOE* and *CYP2D6* variants in AD. Among all the genetic factors that affect the success of AD therapy, *APOE* is certainly the most important and affects over 50% of AD cases [253]. In order to investigate the effects of *APOE* variants on multifactorial treatment, a two-year study with three drugs was conducted. *APOE* 3/4 carriers emerged as the best responders, and *APOE* 4/4 carriers as the worst. The response of *APOE* 2/3, *APOE* 4/4, and 4/5 was similar, where patients, after initial improvement, showed rapid deterioration [254].

Genetic polymorphisms in *CYP2D6* significantly affect drug metabolism and the interindividual response to therapy [255]. The study that investigated the influence of *CYP2D6* variants on the therapeutic response in AD patients used a four-drug therapy protocol for 1 year (Table 3). The results showed that *CYP2D6*- extensive metabolizers and *CYP2D6*- intermediate metabolizers were the best responders to multifactorial therapy with cognition improvement after 1 year period, while in *CYP2D6*- poor metabolizers and CYP2D6- ultra-rapid metabolizers, there was no therapeutic effect and cognitive functions continuously decreased during the mentioned period [256]. Other polymorphic variants, like those of *PS1* and *PS2* genes, can influence the outcome of AD therapy in general and, therefore, multifactorial therapy, as well (Table 3). Patients with different *PS1* variants did not show significant differences in response to therapy, while regarding the *PS2* gene, depending on the exon five variants, responses to therapy differed significantly, and *PS2*− patients responded much better to therapy than those with *PS2*+ [257].

One of the challenges of multifactorial therapy is the existing comorbidities of patients. Due to the relatively late onset of dementia, comorbidities are common and are mostly related to older age. In a study that included 2618 patients with AD, the average age was 76.1 years, and the most common comorbidities were hypertension, osteoarthritis, depression, diabetes mellitus, and cerebrovascular disease [258]. The first problem that comes out of the above is the drug–drug interaction. Medications that a patient is receiving for existing conditions can reverse or modify the effects of dementia therapy and, thus, represent a major obstacle to its effectiveness.

Another problem is the side effects of dementia therapy itself. The AChEIs side effects are associated with enhanced cholinergic tone [259], memantine causes off-target effects in other neurotransmitter systems, and its side effects are related to its anti-glutamatergic activity [260] and even the first disease-modifying treatments for AD, anti-amyloid antibodies such as aducanumab and lecanemab are associated with amyloid-related imaging abnormalities (ARIA), which come in two forms: ARIA-E characterized by edema and ARIA-H characterized by hemorrhage [261,262]. Carriers of the *APOE ε4* allele showed an increased risk for ARIA, with *APOE ε4* homozygotes being more prone to severe ARIA [263]. Although there are no FDA-approved tests to determine individual genetic status prior to anti-amyloid treatment, the recommendation is that AD patients should be pre-screened for *APOE* genotypes due to the risk for ARIA [148,262].

## 7. Pharmacogenomics of Non-Cognitive Symptoms: Antipsychotic, Antidepressant, and Antiepileptic Drugs

Non-cognitive symptoms, i.e., BPSD, are a major contributor to the heterogeneity of dementia. BPSD varies in different stages of the disease and includes symptoms such as depression, anxiety, apathy, agitation, delusions, and hallucinations. Due to their variability and prevalence, corresponding therapeutic approaches for these symptoms are an important part of the treatment of patients with dementia [264]. As demonstrated in Table 4, pharmacological approaches to dementia treatment include, among others, psychotropics (e.g., antipsychotics, antidepressants, anticonvulsants) [265]. The majority of psychotropic drugs used for treating neuropsychiatric diseases are metabolized by CYP1A2, CYP2B6, CYP2C8/9, CYP219, CYP2D6, and CYP3A4 enzymes [265].

Studies have shown that antidepressant drugs, including tricyclic antidepressants, SSRIs, and norepinephrine/serotonin-reuptake inhibitors, are major substrates of CYP1A2, CYP2C9, CYP2C19, CYP2D6, CYP3A4, UGT1A4, and UGT1A3 enzymes, while typical and atypical antipsychotics are the main substrate of CYP1A2, CYP2C19, CYP2D6, CYP3A4, and UGT1A4 enzymes [8,265]. Enzymes that are mostly involved in the metabolism of antidepressants and antipsychotics are CYP2D6 (86% and 72%, respectively) and CYP3A4 (72% and 75%, respectively) [8,265]. Different classes of anticonvulsants, i.e., antiepileptics (benzodiazepines, barbiturates, miscellaneous antiepileptics, fatty acid derivatives, succinimides, oxazolidines, and hydantoin derivatives), are also mostly metabolized by CYP enzymes.

For example, over 65% of antiepileptic drugs are major substrates for CYP (CYP3A4, CYP3A5, CYP2E1, CYP2C8, CYP2B6, CYP2D6, CYP2C19, CYP1A2, CYP2C9, CYP1A1, CYP1A6, CYP3A7, CYP2C18, CYP4B1) or UGT enzymes (UGT1A1, UGT1A3, UFT1A9, UGT2B7, UGT1A4, UGT1A6, UGT1A10, UGT2B15) [254,265,266]. CYP3A4 is involved in the drug metabolism of most psychotropic drugs, compared to other isoforms [265]. CYP2D6 enzyme plays an important role in oxidase reactions for a large number of commonly prescribed antidepressants, antipsychotics, and antiepileptics, which may act as substrates, inducers, or inhibitors [10,218,265]. However, it has been reported that certain enzymatic activities of CYP2D6 and CYP2C19 are associated with treatment discontinuation [267].

More than 100 different *CYP2D6* alleles might show deficient (poor metabolizer, PM), normal (extensive metabolizer, EM), intermediate (intermediate metabolizer, IM), or increased (ultra-rapid metabolizer, UM) enzymatic activity, meaning that different patients will require different dosages [218,268]. While the majority of the general population shows normal enzymatic activity [265,268], the proportion of extensive metabolizers and ultra-rapid metabolizers is slightly higher in the general population compared to AD cases [265]. On the other hand, the proportion of intermediate metabolizers and poor metabolizers is vaguely lower in the general population compared to AD cases.

It has been shown that between 10 and 20% of Caucasians carry defective *CYP2D6* variants that influence drug metabolism, especially the metabolism of psychotropics. For example, it is shown for several antidepressants (amitriptyline, clomipramine, citalopram, doxepin, escitalopram, fluvoxamine, imipramine, paroxetine, sertraline, and trimipramine) and antipsychotics (aripiprazole, brexiprazole, haloperidol, pimozide, risperidone, and zuclopenthixol) that discontinuation of their treatment is associated with deficient and/or increased enzymatic activity of CYP2C19 and/or CYP2D6 [267].

Likewise, a large number of individuals with altered responsiveness to benzodiazepines and neuroleptics show deficient or increased enzymatic activity, i.e., carry mutant variants of the *CYP3A4*, *CYP2D6*, and *CYP2C9* genes [265]. Association studies of *CYP2D6* variants and genes (*ACE*, *AGT*, *APP*, *MAPT*, *APOE*, *PSEN1*, *PSEN2*, *FOS*, and *PRNP*) related to dementia demonstrated that in individuals with deficient or increased enzymatic activity, there is an accumulation of the risk variants, which might influence therapeutic response [265]. Though there were no reported differences between females and males in the general population, the proportion of extensive metabolizers was somewhat higher in females than in males with AD, whereas poor metabolizers were more frequent in males than females with AD, suggesting a higher risk for males of developing an adverse drug reaction [265].

In addition, variations in certain genes are associated with geographic and ethnic differences, which affect drug metabolism and, correspondingly, individual responses to a certain therapeutic approach [265]. Associations between certain genetic variants encoding for various enzymes involved in drug metabolism and the effects (positive or adverse) of drug treatment have been extensively studied. The metabolism of antidepressants, antipsychotics, and anticonvulsants also includes, among others, various groups of enzymes (esterases, transferases, reductases, oxidases, histamine methytransferases), receptors (adrenergic, dopamine, and serotonin receptors), transporters (solute carrier family 6, ATP-binding cassettes), and channels (potassium voltage-gated and sodium channels), which are genetically variable [265,269]. Polymorphic variations in the genes encoding for these proteins may influence drug metabolism [269].

For example, variants in the genes encoding for the transporters of antipsychotics (*ABCB1*, *SLC6A2*, *SLC6A4*, *SCN5A*, *KCNH2*, *KCNE1*, *KCNE2*, *KCNQ1*) and antidepressants (*SLC6A4*, *SLC6A2*, *ABCB1*, and *5-HTTLPR*) influence the metabolism of these drugs [8]. It has also been reported that responsiveness to antipsychotics is higher in Ins/Ins carriers (−141C Ins/ins), as well as in A1 carriers of the Taq 1 A SNP of the *DRD2* gene. The Ser allele of the Ser9Gly SNP in the *DRD3* gene was associated with a better response to clozapine, while the Gly allele was associated with a higher risk for tardive dyskinesia [268]. Several SNPs in the *SLC6A4* and *SLC6A3* genes showed an association between clozapine responsiveness and genotype or allele frequencies [8,266]. In addition, antipsychotic-induced extrapyramidal symptoms (*DRD2*, *HTR2A*, *GRIK3*, *SLC6A4 VNTR*, *COMT Val158Met*, *ADORA1*, *ADORA3*, *ADORA2A*), tardive dyskinesia (*HTR2A*, *HTR2C*, *DRD2*, *DRD3*, *DPP6*, *SOD2*, *CYP2D6*, *CNR1*, *HSPG2*), metabolic syndrome (*HTR2C*, *LEP*, *LEPR*), and other antipsychotic-induced symptoms have been associated with polymorphisms in several genes (*DRD2*, *LEP*, *BDNF*, *LPL*, *TPH*, etc.) [266]. Moreover, Met/Met homozygotes of the Val108Met SNP in the *COMT* gene showed a better response to clozapine [266].

Several polymorphisms in the serotonin receptor gene (*5HTR2A*) showed that certain variants are associated with a better response to clozapine (A/A, A-1438G; His allele, His452Tyr), olanzapine (A/A genotype of A-1438G), or risperidone (C/C, T102C). Moreover, repeat-length polymorphisms in the serotonin transporter gene have been associated with responses to certain antidepressants and antipsychotics. For example, a *long allele* is associated with a better response to citalopram, paroxetine, fluoxetine, risperidone, and clozapine [268], while *CYP2D6* and *CYP2C19* variants are associated with antidepressant-induced symptoms, such as nightmares, anxiety, and panic attacks [8].

**Table 4:** Pharmacogenomics of antipsychotic, antidepressant, and antiepileptic drugs.

Drug Name	Drug Class	Associated Gene	Pharmacogenetics Finding	References
Aripiprazole, Brexiprazole, Risperidone	Atypical antipsychotic	*CYP2C19*, *CYP2D6*	Treatment discontinuation associated with PM/UM enzymatic activity	[267]
Haloperidol, Pimo-zide, Zuclopenthixol	Typical antipsychotic			
Various antipsychotics		*SLC6A4*, *SLC6A2*, *ABCB1*, *5-HTTLPR*	Variants influence antipsychotic metabolism	[8]
Various antipsychotics		*DRD2*	Higher response to antipsychotics in Ins/Ins (−141C Ins/Ins), and A1 carriers (Taq 1 A)	[268]
Clozapine	Atypical antipsychotic	*DRD3*	Ser allele of Ser9Gly associated with better clozapine response	[268]
Clozapine	Atypical antipsychotic	*DRD3*	Gly allele associated with higher risk for tardive dyskinesia	[268]
Clozapine	Atypical antipsychotic	*SLC6A4*, *SLC6A3*	Associated with clozapine response	[8,266]
Various antipsychotics		*DRD2*, *HTR2A*, *GRIK3*, *Val158Met*, *SLC6A4*, *VNTR*, *ADORA1*, *ADORA3*, *ADORA2A*, *COMT*	Associated with antipsychotic-induced extrapyramidal symptoms	[8,266]
Various antipsychotics		*HTR2A*, *HTR2C*, *DRD2*, *DRD3*, *DPP6*, *SOD2*, *CYP2D6*, *CNR1*, *HSPG2*	Associated with antipsychotic-induced tardive dyskinesia	[8]
Various antipsychotics		*HTR2C*, *LEP*, *LEPR*	Associated with antipsychotic-induced metabolic syndrome	[8]
Various antipsychotics		*DRD2*, *LEP*, *BDNF*, *LPL*, *TPH*	Associated with antipsychotic-induced other symptoms	[8]
Clozapine	Atypical antipsychotic	*COMT*	Better clozapine response in Val108Met Met/Met homozygotes	[268]
Clozapine, Olanzapine, Risperidone	Atypical antipsychotic	*5HTR2A*	Variants associated with better response to clozapine (A/A, A-1438G; His allele, His452Tyr), olanzapine (A/A, A-1438G), or risperidone (C/C, T102C)	[268]
Clozapine Risperidone	Atypical antipsychotic	*SLC6A4*	Long allele associated with better risperidone and clozapine response	[268]
Various antipsychotics		*CYP2D6*, *ACE*, *AGT*, *APP*, *MAPT*, *APOE*, *PSEN1*, *PSEN2*, *FOS*, *PRNP*	PM or UM enzymatic activity influence therapeutic response	[265]
Amitriptyline, Clomipramine, Doxepin, Imipramine, Trimipramine	Tricyclic antidepressant	*CYP2C19*, *CYP2D6*	Treatment discontinuation associated with PM/UM enzymatic activity	[267]
Citalopram, Escitalopram, Fluvoxamine, Paroxetine, Sertraline	Selective serotonin reuptake inhibitor			
Various antidepressants		*ABCB1*, *SLC6A2*, *SLC6A4*, *SCN5A*, *KCNH2*, *KCNE1*, *KCNE2*, *KCNQ1*	Variants influence antidepressant metabolism	[8]
Paroxetine, Citalopram, Fluoxetine	Selective serotonin reuptake inhibitor	*SLC6A4*	Long allele associated with better response to citalopram, paroxetine, fluoxetine	[268]
Various antidepressants		*CYP2D6*, *CYP2C19*	Variants associated with antidepressant-induced nightmares, anxiety, panic attacks	[8]
Valproic acid	Fatty acids	*GRIN2B*	200T>G allele carriers require lower dose of valproic acid	[266]
Valproic acid	Fatty acids	*UGT1A6*	Carriers of 541A>G, 552A>C, and 19T>G alleles need higher dose of valproic acid	[266]
Carbamezapine	Carboxamides	*SCN1A*, *ABCB1*, *UGT2B7*, *ABCC2*, *CYP1A2*, *HNF4A*, *CYP3A5*	Associated with altered carbamazepine metabolism	[266]
Phenytoin	Hydantoins	*SCN1A*, *CYP2C9*, *CYP2C19*, *ABCB1*	Variants influence phenytoin metabolism	[266]
Clobazam	Benzodiazepine	*CYP2C19*, *CYP3A4*, *CYP3A5*	Some genotype carriers prone to adverse clobazam reactions	[8]

PM—poor metabolizer; UM—ultra-rapid metabolizer.

Furthermore, polymorphisms in the genes *SCN1A*, *ABCB1*, *UGT2B7*, *ABCC2*, *CYP1A2*, *HNF4A*, and *CYP3A5* are associated with altered drug metabolism of carbamazepine. Certain variants in the genes *SCN1A*, *CYP2C9*, *CYP2C19*, and *ABCB1* influence the metabolism of phenytoin [266]. Moreover, pathogenic variants in the *SLC2A1* gene might predict the responsiveness and selection of adequate antiepileptics [8]. Clobazam is a substrate for several CYP enzymes (CYP2C19, CYP3A4, CYP2B6, CYP2C18), while individuals with certain genotypes in the *CYP2C19*, *CYP3A4*, and *CYP3A5* genes require adjustments in clobazam dosage due to adverse drug reactions [8].

According to the different genes involved in the pharmacogenomics of AD as well as the response to antipsychotics, antidepressants, and antiepileptics, further studies are necessary for better characterization of the pharmacogenomics profile and determination of drug efficacy and safety in the treatment of non-cognitive symptoms of AD [5].

## 8. Pharmacogenomics of Non-Cognitive Symptoms: Anxiolytic, Hypnotic, and Sedative Drugs

Excessive anxiety and worry, as well as restlessness, fatigue, concentration problems, irritability, muscle tension, and sleep disturbance, are common symptoms in patients with dementia. According to a recent meta-analysis [270], prevalence rates of anxiety in dementia are around 40%, with no obvious association with the stages of illness or dementia severity [271]. Moreover, people with dementia often experience sleep problems such as insomnia, impaired nocturnal sleep with increased awakenings, and decreased rapid eye movement (REM) sleep, as well as increased daytime sleep [272,273]. The prevalence of sleep disorders especially rises in patients with VaD, LBD, or dementia related to PD [273]. Additionally, sleep disruption normally interferes with the maintenance of cognitive health and is associated with the rate of cognitive decline in older adults [274].

Anxiolytics, hypnotics, and sedatives are pharmaceuticals used for a reduction in anxiety, to relieve sleep difficulties, or to induce a calming effect. The primary group of medications within this category includes benzodiazepines. They are one of the most prescribed pharmaceuticals in developed countries, commonly used for the treatment of anxiety, sleep disorders, agitation, and alcohol withdrawal [275]. However, the treatment methods for these non-cognitive disorders are more challenging in the context of dementia because, in dementia, they can manifest differently than in typical early-onset individual disorders. Moreover, benzodiazepines, as first-line anxiolytics and commonly used sedatives, might contribute to cognitive and psychomotor impairment [276]. Due to the extensive list of potential side effects, the use of psychotropic drugs in older patients with dementia must be individually tailored. This means that, in addition to comorbidities and other concomitant medicines, distinctive individual characteristics, including pharmacogenomics factors, should be addressed when estimating the risks and benefits of prospective therapy.

Metabolism of most benzodiazepines starts with oxidation, followed by conjugation to glucuronide, which is then eliminated by the urine [277]. Although there are benzodiazepines that are directly conjugated, most of them go through the oxidation stage catalyzed by liver CYP enzymes [278], whose activity greatly influences drug metabolism and plasma concentration. As already mentioned, genes coding for CYP enzymes are highly polymorphic, influencing the enzyme’s activity and leading to absent, reduced, or increased drug metabolism. Consequently, higher drug concentrations, due to the poor metabolizing ability, can increase side effects or toxicity, while on the other hand, due to extensive drug metabolism, efficient therapeutic doses can be higher than usual.

The majority of benzodiazepines are metabolized by CYP2C19 and CYP3A4/5; other enzymes such as CYP1A2, CYP2C9, and CYP2B6 may also play a role in the metabolism of some benzodiazepines [279,280]. For example, it is known that diazepam is metabolized to nordiazepam by CYP2C19 and CYP3A4 and to temazepam by CYP3A4. Both metabolites undergo hydroxylation to oxazepam, which is catalyzed by CYP3A4 and/or CYP2C19 [279]. However, a recent paper showed that the CYP2B6 phenotype also affects diazepam pharmacokinetic variability [281]. Additionally, it was shown among the elderly population that carriers of CYP2C9*2 and CYP2C9*3, as poor metabolism alleles, have an increased risk of falls associated with diazepam treatment [282]. There are more than 40 polymorphic variants of the CYP2C19 gene, resulting in around 35 enzyme isoforms [283] with at least 7 alleles (CYP2C19*2 to CYP2C19*8) associated with partial or complete inactivation of the enzyme resulting in poor drug metabolism [279]. On the other hand, the CYP2C19*17 variant is associated with increased activity, and carriers of this allele, especially homozygotes, are considered extensive metabolizers [284]. The presence of poor metabolism alleles raises the chance of diazepam side effects, whereas the presence of CYP2C19*17 minimizes the risk of side effects but possibly decreases its efficacy when administered in a standard dose [285,286,287]. Since the clearance of benzodiazepines decreases as the number of low metabolizing *CYP2C19* alleles increases [288,289], it would be advisable to adjust their dose according to the *CYP2C19* genotype. For example, Zubiaur et al. [281] recommend lowering the dose of diazepam for 25–50% in patients whose genotype indicates poor drug metabolism.

Enzyme CYP2C19 is also included in the metabolism of clobazam. It was shown that the response to clobazam was higher among carriers of poor metabolism variants, with an evident gene–dose effect [290]. The same trend was noticed in the occurrence of side effects, such as drowsiness and dizziness, which were more prominent in poor metabolizers [290]. Poor CYP2C19-associated metabolism of clobazam in a patient receiving a standard therapeutic dose for seizure disorder caused comatose condition due to the elevated concentration of clobazam active metabolite, norclobazam [291]. Additionally, Riva et al. [292] reported an increased enzymatic activity associated with the CYP2C19*17 allele. They found, however, that the magnitude of observed effects was smaller than the one reported for poor metabolizing alleles, implying that the effects of CYP2C19*17 probably do not have clinical significance, except for medicines with very narrow therapeutic windows [293].

Another benzodiazepine, midazolam, is highly metabolized by CYP3A4 and CYP3A5, and it is also used as a probe substrate in studying the activity of those enzymes [294]. Amino acid sequences for the two enzymes have 83% similarity, and the main differences between them are in their active sites and substrate access channels [295]. There are studies reporting the association between *CYP3A5* genotype and rates of midazolam hydroxylation [296] and clearance [297]. However, it seems that CYP3A genetics has only a limited impact on midazolam metabolism in vivo. Specifically, several studies reported a lack of the functional significance of polymorphisms resulting in common variants, including CYP3A4*1B, CYP3A5*3, CYP3A5*6, and CYP3A5*7 [298,299,300]. This could be due to the fact that midazolam is also a highly permeable substrate of P-glycoprotein [301]. Additionally, plasma midazolam concentration and sedation grade were found to be associated with 1236C>T polymorphism of the *MDR1* (multidrug resistance 1) gene [302].

Metabolism of lorazepam, as well as structurally related benzodiazepines oxazepam and temazepam, skips phase I catalyzed by the CYP enzymes and is predominantly based on glucuronidation [303]. Enzymes included in pharmaceuticals’ glucuronidation are uridine 5′-diphosphate-glucuronosyltransferases (UGTs) [304], with UGT1 and UGT2 enzymes mostly involved in drug metabolism processes. Variations in their genes, resulting in changes in their expression and function, are significant contributing factors to interindividual variability in drug disposition [305]. For instance, the *UGT2B15* genotype highly affects the pharmacokinetics of lorazepam. A single nucleotide polymorphism (G/T) in *UGT2B15* gene coding region can result in UGT2B15*2 variant, which is associated with lower systemic clearance and metabolic activity of lorazepam and significantly higher lorazepam concentrations in homozygotes [306]. Higher lorazepam plasma levels are associated with more pronounced clinical effects. For example, it was shown that UGT2B15*2 homozygotes, especially women, have greater postoperative anxiety reduction after lorazepam premedication when compared with carriers of other genotypes [307].

Structurally different from benzodiazepines but with a similar mechanism of action via GABA signaling are Z-drugs, which have significant hypnotic effects by reducing sleep latency and enhancing sleep quality [308]. The major metabolism pathways of Z-drug zolpidem include hydroxylation followed by oxidation, mediated mostly by CYP3A4; however, CYP2C9, CYP1A2, CYP2D6, and CYP2C19 have also been reported to be included [309]. In a previous study, the CYP3A4*18 variant was associated with increased and CYP2C19*2 with reduced zolpidem metabolism [310]. However, other authors reported no evidence for the impact of the *CYP2C19* genotype on the pharmacokinetic parameters of zolpidem [311]. In another study, participants received zolpidem and clarithromycin, a CYP3A4 inhibitor, in order to eliminate the contribution of CYP3A4 to zolpidem metabolism. However, no differences in zolpidem plasma concentrations were found when subjects were divided according to *CYP2D6* genotype [312]. Similarly, a lack of association between *CYP2C9* genotype and zolpidem metabolism was also reported [313]

Pharmacogenomic research resulted in various findings contributing to the improvement in establishing the anxiety treatment and predicting its outcome (Table 5). It is obvious that both clinicians and patients could benefit from defining the relations between genetic variation and variable drug responses to anxiolytics and sedatives, especially considering the high prescription rates of this group of psychiatric medications.

## 9. Conclusions

So far, dementia treatment has been directed against only several pharmacological targets, emphasizing the need for the development of novel therapeutic strategies. Since the therapeutic response is a complex trait, it is not likely that a single drug could be effective in the treatment of a variety of cognitive impairments, behavioral disturbances, and functional decline [4]. Therefore, multifactorial treatments with a combination of several drugs represent the most feasible option in dementia. However, current as well as potential novel anti-dementia treatments of both cognitive and neuropsychiatric symptoms require evaluation from a pharmacogenomic perspective on a case-by-case basis in order to obtain optimal therapeutic efficacy, as well as to avoid drug side effects and unnecessary costs. Pharmacogenomics could offer help in detecting safer and more effective medications for each dementia patient, as well as new pharmacotherapeutic targets, whose identification has been complicated by the interplay of numerous genetic factors with only minor, moderate effect on pharmacokinetic or pharmacodynamic variability [314]. Although prediction of drug response with respect to genetic variations affecting ADME has already been established, further studies are needed to better understand the functional consequences of genetic polymorphisms in neurotransmitter receptors, transporters, and signal transduction molecules.

In addition to genetic background, drug efficacy, and safety are influenced by many other factors, including mechanisms of drug action, drug-specific adverse reactions, drug–drug interactions, nutritional factors, etc. [265]. Patient characteristics, such as age, gender, and ethnicity, also represent important parameters that might determine individual drug response. Despite the accumulation of genetic information on dementia, the role of epigenetic and environmental factors is still not well known. Hence, in order to better understand such complex multifactorial disorders, both gene–gene and gene–environment models need to be established. Moreover, recent progress in functional genomics, proteomic profiling, high-throughput screening methods, large databases, and bioinformatic tools stimulates the development of pharmacogenomic studies, speeding up clinical trials, improving patient stratification, reducing costs and potential adverse effects and optimizing therapeutic outcomes [2]. Despite the challenge of translation from the research laboratory into clinics, pharmacogenomics holds promise of future cost-effective, safe, and efficacious personalized medicine for patients with dementia. However, future research and strategy advances are needed to overcome scientific, economic, and clinical obstacles and involve pharmacogenomics as a routine intervention in personalized treatment approaches in neuropsychiatry worldwide.

## Figures and Tables

**Table 1:** Genetics of most common types of dementia.

Dementia	Prevalence	Prevalence of Sporadic Cases	Highly Associated Genes	Other Involved Genes
AD	60–80%	95–90%	Early-onset: *APP*, *PSEN1*, *PSEN2* Late-onset: *APOE*	*CR1*, *BIN1*, *ADAM10*, *SORL1*, *SIRT1*, *BACE1*, *TREM2*, *AMY1A*, *ABCA7*, *TOMM40*, *SULTA3/4*
VaD	15%	Mostly sporadic	VaD due to monogenic disorders: *NOTCH3*, *HTRA1*, *GLA*, *APP*, *PSEN1*, *PSEN2*, *COL4A1*	*APOE*, *MTHFR*, *PON1*, *RPGRIP1L*, *PHLDB2*, *SYK*
FTD	2.7% (total population) 10.2% (younger population)	70%	*C9ORF72*, *MAPT*, *GRN*	*TBK1*, *VCP*, *OPTN*, *TARDP*, *CHMP2B*, *TREM2*, *UBQLN2*, *SQSTM1*, *FUS*, *LOC730100*, *CEP131*, *ENTHD2*, *C17orf89*, *CHCHD10*, *SIGMAR1*, *CCNF*, *TIA1*
LBD	4.2% (total population) 7.5% (older population)	>80%	*APOE*, *SNCA*, *GBA*	*BIN1*, *TMEM175*, *CLU*, *FBXL19*, *MAPT*, *TPCN1*, *OPTN*

**Table 3:** Multifactorial therapy related to *APOE*, *CYP2D6*, *PS1*, and *PS2* genes.

Gene	Multifactorial Therapy Protocol	Study Length	Responders (Best to Worse)	References
*APOE*	CDP-choline (1000 mg/day) piracetam (2400 mg/day) anapsos/calagualine (360 mg/day)	2 years	*APOE*-3/4 > *APOE*-2/3 > *APOE*-3/3 > *APOE*-2/4 > *APOE*-4/4	[257]
*CYP2D6*	CDP-choline (500 mg/day) piracetam (1600 mg/day) nicergoline (5 mg/day) donepezil (5 mg/day)	1 year	EM > IM > PM > UM	[256]
*PS1*	CDP-choline (1000 mg/day) piracetam (2400 mg/day) anapsos/calagualine (360 mg/day)	2 years	*PS1*-2/2 = *PS1*-1/2 > *PS1* 1/1	[257]
*PS2*	CDP-choline (1000 mg/day) piracetam (2400 mg/day) anapsos/calagualine (360 mg/day)	2 years	*PS2*− > *PS2*+	[257]

PM—poor metabolizer; IM—intermediate metabolizer; EM—extensive metabolizer; UM—ultra-rapid metabolizer.

**Table 5:** Pharmacogenomics of anxiolytic, hypnotic, and sedative drugs.

Drug Name	Drug Class	Associated Gene	Pharmacogenetics Finding	Reference
Diazepam	Benzodiaze-pine, GABA-A receptor agonist	*CYP2C19*	Variants CYP2C19*2 to CYP2C19*8 associated with partial or complete inactivation of enzymes, resulting in poor drug metabolism	[279]
			CYP2C19*17 variant associated with increased enzyme activity (extensive metabolizers)	[284]
			CYP2C19*2 allele raises risk of side effects, whereas CYP2C19*17 minimizes risk of side effects but decreases its efficacy	[285,286]
			Clearance decreases as number of low metabolizing CYP2C19 alleles increases	[281,289]
		*CYP2B6*	*CYP2B6* genotype affects diazepam pharmacokinetic variability	[281]
		*CYP2C9*	CYP2C9*2 and CYP2C9*3 alleles associated with poor diazepam metabolism and increased risk of falls among the elderly population	[282]
Clobazam	Benzodiazepine, GABA-A receptor agonist	*CYP2C19*	Response rate to clobazam and occurrence of side effects are higher among carriers of CYP2C19*2 and CYP2C19*3 variants, with evident gene–dose effect	[290,291]
			CYP2C19*17 allele associated with increased enzymatic activity, but magnitude of observed effects is smaller than one for poor metabolizing alleles	[292]
Midazolam	Benzodiazepine, GABA-A receptor agonist	*CYP3A5*	Mean clearance is lower in CYP3A5*3 allele carriers	[297]
			Limited or no functional significance of polymorphisms resulting in common variants, including CYP3A4*1B, CYP3A5*3, CYP3A5*6, and CYP3A5*7	[298,299,300]
		*MDR1*	Plasma concentration and sedation grade associated with *MDR1* 1236C>T SNP	[302]
Lorazepam	Benzodiazepine, GABA-A receptor agonist	*UGT2B15*	UGT2B15*2 variant associated with lower systemic clearance and metabolic activity of lorazepam and higher lorazepam concentrations in homozygotes	[306]
			UGT2B15*2 homozygotes, especially women, have greater postoperative anxiety reduction after lorazepam premedication	[307]
Zolpidem	Imidazopyridine, GABA-A receptor agonist	*CYP3A*	CYP3A4*18 variant associated with increased zolpidem metabolism	[310]
		*CYP2C19*	CYP2C19*2 variant associated with reduced zolpidem metabolism	[310]
			No effect of *CYP2C19* genotype on pharmacokinetic parameters of zolpidem	[311]
		*CYP2D6*	No effect of *CYP2D6* genotype on pharmacokinetic parameters of zolpidem	[312]
		*CYP2C9*	No effect of *CYP2C9* genotype on pharmacokinetic parameters of zolpidem	[313]
